# Metabolic engineering of the chloroplast genome reveals that the yeast *ArDH* gene confers enhanced tolerance to salinity and drought in plants

**DOI:** 10.3389/fpls.2015.00725

**Published:** 2015-09-09

**Authors:** Muhammad Sarwar Khan, Benish Kanwal, Shahid Nazir

**Affiliations:** ^1^Center of Agricultural Biochemistry and Biotechnology, University of Agriculture, FaisalabadPakistan; ^2^National Institute for Biotechnology and Genetic Eningeering, FaisalabadPakistan; ^3^Agricultural Biotechnology Research Institute – Ayub Agricultural Research Institute, FaisalabadPakistan

**Keywords:** chloroplast transformation, arabitol dehydrogenase, salt and draght tolerance, osmoprotectants

## Abstract

Osmoprotectants stabilize proteins and membranes against the denaturing effect of high concentrations of salts and other harmful solutes. In yeast, arabitol dehydrogenase (*ArDH*) reduces D-ribulose to D-arabitol where D-ribulose is derived by dephosphorylating D-ribulose-5-PO4 in the oxidized pentose pathway. Osmotolerance in plants could be developed through metabolic engineering of chloroplast genome by introducing genes encoding polyols since chloroplasts offer high level transgene expression and containment. Here, we report that *ArDH* expression in tobacco chloroplasts confers tolerance to NaCl (up to 400 mM). Transgenic plants compared to wild type (WT) survived for only 4–5 weeks on 400 mM NaCl whereas plants remained green and grew normal on concentrations up to 350 mM NaCl. Further, a-week-old seedlings were also challenged with poly ethylene glycol (PEG, up to 6%) in the liquid medium, considering that membranes and proteins are protected under stress conditions due to accumulation of arabitol in chloroplasts. Seedlings were tolerant to 6% PEG, suggesting that ARDH enzyme maintains integrity of membranes in chloroplasts under drought conditions via metabolic engineering. Hence, the gene could be expressed in agronomic plants to withstand abiotic stresses.

## Introduction

Crop production is severely affected by the accumulated salts in the soil. For example, about 20% of the world’s agricultural soils are affected by salinity (Zhu, 2001) and about 25% of US soils are subjected to drought (Boyer, 1982; Tanji, 1990; Zhu, 2001). The damaging effects of salts on plants are mainly because of two reasons; one, water deficit resulting in osmotic stress and two, ions stress to biochemical processes (Ward et al., 2003). To withstand such osmotic stresses, plants synthesize, and accumulate compatible solutes, commonly known as osmoprotectants. These molecules stabilize proteins, membranes, and even transcriptional and translational machineries in the cells against the denaturing effect of accumulated salts and other damaging solutes (Yancey, 1994; Wani et al., 2013). The physicochemical basis of the osmoprotective effect of osmolytes requires the exclusion of osmoprotectants from the hydration sphere of proteins (Timasheff, 1992). Under this situation, structures of the native proteins are thermodynamically favored as they offer minimal surface area to the water (McNeil et al., 1999; Rontein et al., 2002). Whereas most salts interact directly with the protein surfaces, causing denaturation of proteins. It means under stressful conditions these osmoprotectants can advance cellular osmotic pressure and protect constituents of cells.

Osmoprotectants, based on their chemical properties, are of three types: betaines; amino acids like proline and non-reducing sugars such as trehalose and arabitol. All of these types do not occur in crop plants, however, their beneficial effects are generally not species-specific. Increasing crop tolerance by engineering plant metabolic pathways is one of the candidate approaches (LeRudulier et al., 1984; Sakamoto and Murata, 2001). Majority of plants cannot metabolize most sugar alcohols including D-arabitol (Stein et al., 1997) hence, a gene that encodes an enzyme for developing sugar alcohol can be expressed via plant chloroplast genome to provide osmoprotection under stress regimes.

Chloroplast transformation compared with conventional transgenic technologies has the unique advantages of very high level gene expression and accumulation of expressed proteins and increased transgene containment. In most cultivated plant species the chloroplasts are inherited uniparentally in a strictly maternal fashion thereby greatly reduce the risk of genetic outcrossing. Therefore, incorporating a gene conferring salt and/or drought tolerance will not call upon the question of potential drought resistance development in wild-type species. For example, chloroplast transgenic plants expressed very high level of transprotein (McBride et al., 1995; Khan and Maliga, 1999; Kota et al., 1999; Viitanen et al., 2004) due to polyploid nature of plastids and engineered translation control elements (Eibl et al., 1999; Kuroda and Maliga, 2001). Similarly, chloroplast derived herbicide resistance and salt tolerance overcome outcross problems of nuclear transformation because of strict maternal inheritance of plastid genomes (Daniell et al., 1998, 2002; Scott and Wilkinson, 1999; Lee et al., 2003; Khan et al., 2005). Currently, plastid transformation technology is regarded as one of the best approaches to express pharmaceuticals and vaccines with biological activity (Daniell and Khan, 2003; Fernandez-San Millan et al., 2003; Tregoning et al., 2003; Magee et al., 2004). This study has been undertaken to engineer plastid genome for stress tolerance through accumulating a non-reducing plant sugar, arabitol in tobacco plants. The spectinomycin-resistant plants were developed in the laboratory of the corresponding author and were analyzed for transgene integration into the plastome (Plastid genome). Later on, an M Phil student (Sarwar, 2007) was given a task to analyze the putative transgenic plants where preliminary studies were carried out to optimize conditions for enzyme and stress assays (Sarwar, 2007, 2013). Encouraged from preliminary results the putative transgenic plants were purified to homoplasmic level through sequential rounds of regeneration on spectinomycin-containing medium. Regenerated plants were subjected to genetic, enzyme, and stress analyses.

Here, we report overexpression of yeast *ArDH* gene in chloroplasts that confers tolerance to salt and drought at high levels than earlier reports. Further, protecting effects of arabitol on chloroplast proteins and membranes under stressful conditions are provided with reduced risk of outcrossing. To the best of our knowledge this is the first report on engineering stress tolerance through alterations in chloroplast metabolic pathway using *ArDH* gene in plants.

## Results

### Development of Transformation Vectors and Transgenic Plants

**FIGURE 1:**
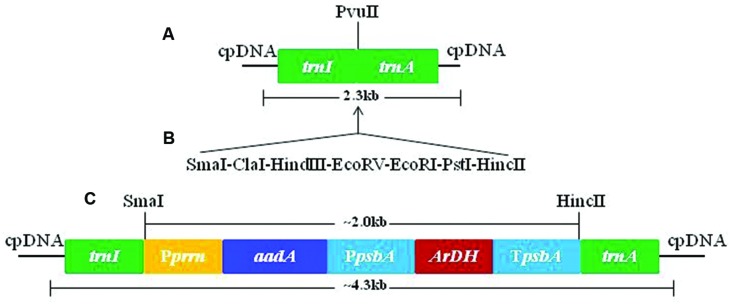
**Sequential amplification, cloning, and development of pMSK83 chloroplast transformation vector. (A)** Tobacco flanks used for site-specific integration of transgenes. **(B)** Adopter was used to create multiple cloning sites to facilitate cloning. **(C)** Final tobacco chloroplast transformation vector with *aadA* and *ArDH* genes.

The chloroplast transformation vector pMSK83 was developed by a sequential process of amplification of DNA fragments from tobacco plastome regions and cloning in pTZ57 plasmid (Fermentas, Germany). The promoter was amplified from 16S rRNA operon to control *aadA* gene expression in chloroplasts (Khan et al., 2007). However, *ArDH* gene was tethered with a light regulated *psbA* promoter along with 5′ and 3′ untranslated regions (UTRs). The *psbA* in chloroplasts is light regulated and hence can be useful in transgene regulation at high levels. The *aadA* gene that confers resistance to both spectinomycin and streptomycin is used for selection of transformation events (Svab and Maliga, 1993; Khan and Maliga, 1999). The *ArDH* gene-containing expression cassette was cloned downstream of *aadA* gene in the chloroplast transformation vector (**Figure 1**). Hence, *aadA* gene is expressing without terminating sequences.

Fully expanded leaves from 6 to 8 weeks old tobacco plants were subjected to bombardments. After 48 h the bombarded leaves were chopped into 3 mm × 3 mm size pieces, which were then placed on spectinomycin (500 mg/l)-containing RMOP medium (Svab and Maliga, 1993; Khan and Maliga, 1999; Khan et al., 2007). Spectinomycin-resistant green shoots started appearing within 4–6 weeks of bombardments from bleached leaf sections on regeneration medium. The green shoots were transferred onto selective maintenance medium for proliferation. To purify the transplastomes to a genetically stable homoplasmic state, leaves from spectinomycin resistant plants were subjected to subsequent rounds of selection and regeneration. During the period the chloroplasts (both the WT and the transformed) and the plastome (the chloroplast genome both WT and the transformed) copies gradually sorted out. Homoplasmic shoots were recovered from leaf sections placed on regeneration medium with or without spectinomycin. From 35 bombardments, nine transgenic clones were recovered on selection and regeneration medium. These plants were rooted on MS medium and subjected to genetic analysis and seed setting for various experiments.

### Genetic Analysis Confirming Stable Integration of Transgene in to Plastome

**FIGURE 2:**
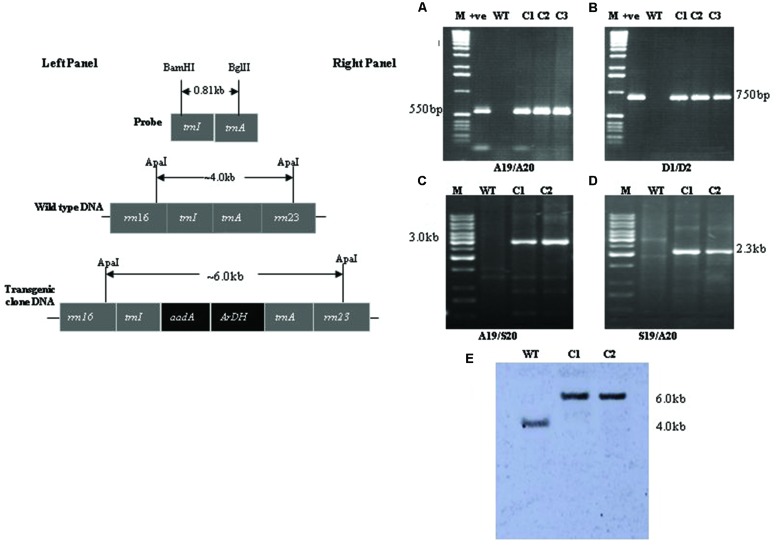
**PCR confirmation of transgene integration and homoplasmy: left; shown is physical map of the probe, flanking sequences and the final transformation vector with important restriction sites.** Right **(A,B)** Amplification of marker gene with primer sets A19/A20 and D1/D2: lane M is 1 kb DNA ladder, lane +ve is plasmid DNA as PCR control, lane WT wild type tobacco DNA, lanes C1, C2, and C3 are transgenic plants DNA **(C,D)** plastome confirmation using primer sets A19/S20 and S19/A20: lane M is 1 kb DNA ladder, lane wild type (WT) tobacco DNA, lanes C1 and C2 are transgenic plants DNA **(E)** Confirmation of homoplasmic status of transplastomics: lane M is 1 kb DNA ladder, lane WT wild type tobacco DNA, lanes C1 and C2 are transgenic plants DNA.

Different sets of primers were used to analyze transgenic plants harboring *aadA* and *ArDH* genes under the control of chloroplast regulatory sequences (Khan et al., 2007). Spectinomycin-resistant plants were analyzed using *aadA* and flanking sequence-specific primers for two reasons; one, confirming the integration of *ArDH* and *aadA* genes into the inverted repeat region. Two, to find out whether the resistance to spectinomycin was due to a mutation in the 16S rRNA gene (Fromm et al., 1987; Svab and Maliga, 1991) or due to the integration of transgenes into the nuclear genome through illegitimate recombination. A primer pair S19 and S20 recognizes sequences that flank the sites of integration on chloroplast genome (**Figure 2**, left). The primers A19 and A20 anneal to the *aadA* gene (selectable marker gene) and the primers D1 and D2 to *ArDH* gene and amplify fragments of 550 and 750 bp, respectively, (**Figures 2A,B**, right). The amplification of 3.0 kb fragment with primer pair A19 and S20 (**Figure 2C**, lanes C1,C2), and 2.3 kb with primer pair S19 and A20 (**Figure 2D**, lanes C1,C2) and absence of fragment from a non-transformed wild type (WT) plant confirms that the spectinomycin-resistant plants carry the *aadA* and *ArDH* genes in the inverted repeat region of the plastome because primers S19 and S20 are specific to inverted repeat regions of the plastome. As chloroplast transformation vector integrates the transgene cassettes into the inverted repeat regions; hence, to test the homoplasmy of the transplastome the sequences used as flanks were used as a probe. The presence of a single hybridizing fragment of ~6.0 kb in Southern blots confirms the homoplasmy of the transplastome, the plastome carrying *aadA* gene and the *ArDH* gene cassettes in the inverted repeats, compared with WT plant where only a 4.0 kb fragment is hybridized with the same probe, reflecting the plastome without insertions of transgenes.

### Qualitative Analysis of *ArDH* Activity in Transgenic Plants

**FIGURE 3:**
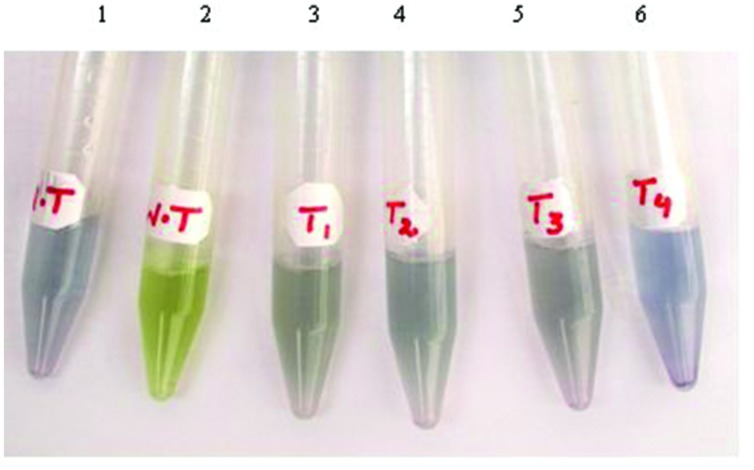
**Color-based detection of *ArDH* gene expression in plant cells.** Tubes 1-2 are leaf extracts from WT plants with (tube 1) and without (tube 2) adding ARDH enzyme in the samples. Tubes 3-6 are transgenic plants that harbor *ArDH* gene in the transplastomes.

Arabitol dehydrogenase enzyme reduces NAD to NADH in the presence of arabitol in the solution and blue color is developed. The crude extracts prepared by homogenizing 0.25 g leaf tissue in 500 μl homogenization buffer, as described in Section “Materials and Methods,” developed blue color depending upon the concentration of the expressed enzyme in the samples from transplastomic plants, confirming the expression of the transgene in the plastid genome of tobacco. Nonetheless, no blue color development was detected in the leaf extracts from WT plants, entrusting original color (**Figure 3** Sample No.2). The colorimetric test was repeated after every 4 weeks and color development was observed in all selected (T1, T2, T3, and T4; T stands for Transgenic) plants. Of these plants T4 exhibited deep color development. Therefore, clone T4 was multiplied and four plants namely; T4-1, T4-2, T4-3, and T4-4 were selected to measure the enzyme activity along with heteroplasmic plants.

### Quantitative Analysis of *ArDH* Activity in Transgenic Plants

**FIGURE 4:**
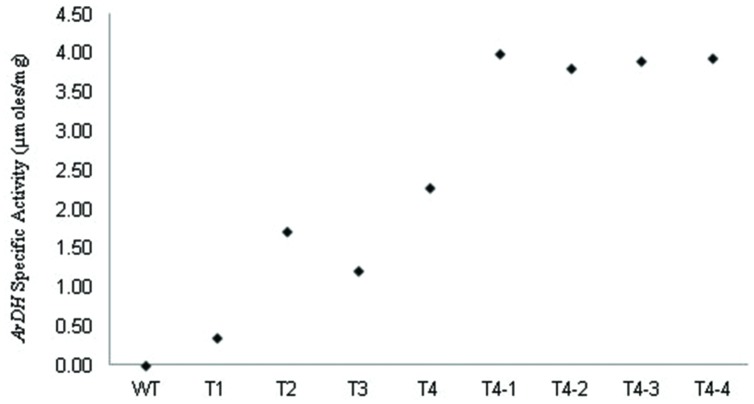
**Levels of ARDH measured in heteroplasmic (T1, T2, and T3) and homoplasmic (T4; T4-1, T4-2, T4-3, and T4-4) plants under standard and continuous light.** Values shown represent the average value of the specific activity recorded in extracts from fourth leaf (from top to bottom) of tobacco plants. Values given for WT, T1, T2, T3, and T4 are from leaf extracts of plants grown under standard growth conditions (100 μmole photons m^-2^ s^-1^ – 16 h light, 8 h dark) at 25°C ± 02. Nevertheless, plants T4-1, T4-2, T4-3, and T4-4, which were regenerated from leaves of T4 transgenic plant were kept under continuous light for 7 days in pots. The enzyme activity recorded for T4-1, T4-2, T4-3, and T4-4 plants is not an average value as they are derived from same T4 clone.

Arabitol dehydrogenase activity was measured in crude extracts of leaves from wild-type and transgenic plants as previously described for yeast by Wong et al. (1995) and on heteroplasmic tobacco seedlings (Sarwar, 2007, 2013). The rate of reduction of NAD to NADH by ARDH enzyme in the presence of D-arabitol was measured by the reduction of MTT (3, 5 dimethylethiazol -2-yl 2, 5 dimethyle tetrazolium bromide) in the solution. Crude extracts from fully expanded leaves (Fourth from top to bottom) of transgenic chloroplast plants showed enzyme activity compared to non-transformed leaves (**Figure 4**). Enzyme activity was different in different plants (T1, T2, T3, and T4 samples) due to their heterogenetic nature though given readings are an average of three samples. The high ARDH enzyme activity was observed in fully expanded leaves of potted plants (T4 homoplasmic transgenic plants) compared to primary heteroplasmic transgenic plants, as described (Sarwar, 2007, 2013). This was due to two reasons: first, copy number for transgene was increased toward homoplasmy. Homoplasmic plants were recovered through multiple rounds on regeneration medium supplemented with spectinomycin (500 mg/L). Second, plants were subjected to analysis after the onset of light as *psbA* 5′ UTR is light regulated (Staub and Maliga, 1994; Eibl et al., 1999). Hence, enzyme activity was enhanced under continuous light yet these levels are very low as expected this may be because of minimal level of substrate availability in chloroplasts.

### Salt Tolerance in Transplastomic Plants

**FIGURE 5:**
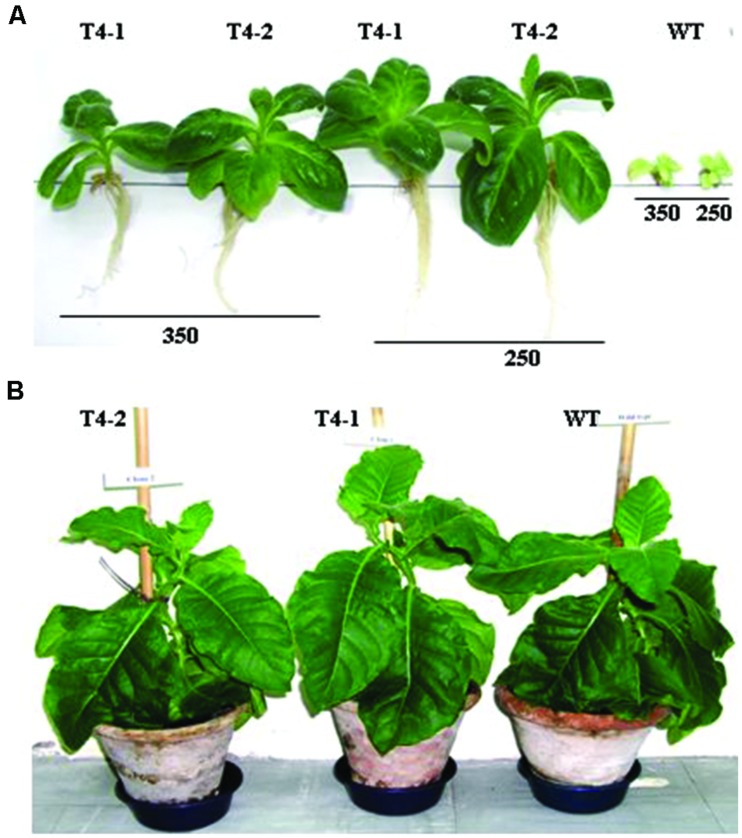
**Salt tolerance assays are performed on *ArDH* gene expressing transplastomic plants on MS medium that carries increasing levels (100–600 mM) of NaCl. (A)** Shows growth of representative plants from T4 clone at 250 and 350 mM concentrations of NaCl whereas in **(B)** plants are growing in pots with indistinguishable phenotypes.

First generation heteroplasmic plants were grown in pots for seed setting and variable degrees of tolerance to NaCl were observed (Sarwar, 2007, 2013) hence, primary transgenic plants growing under *in vitro* conditions were subjected to a number of selection and regeneration rounds to purify transplastomes to homoplasmic level since the process requires 16–17 cell divisions (Moll et al., 1990; Khan and Maliga, 1999). Consequently, confirmed homoplasmic plants were recovered and grown in pots for seed setting. Nodal segments and seeds from homoplasmic transgenic and WT plants were subjected to various levels of NaCl. Plants developed from nodal segments were heterogeneous in growth hence were not used in salt tolerance assays, may give rise to inconsistent results. Therefore seeds of transgenic and WT plants were only used in subsequent assays. Seeds from transgenic plants were placed on solidified MS medium (Murashige and Skoog, 1962) supplemented with increasing concentrations (100–600 mM) of NaCl in plastic Petri plates along with seeds of WT plant in growth room under standard temperature and light conditions. After attaining a reasonable height the plants were transplanted into magenta boxes at same levels of NaCl-containing solidified MS medium. Transgenic plants (T4-1 and T4-2) expressing the *ArDH* gene thrive well up to 350 mM NaCl (**Figure 5A**), whereas WT plants exhibited retarded growth with yellow phenotype, indicating that chloroplast-based expression of ARDH is adequate to confer high level of salinity tolerance in plants. Both transgenic and WT plants developed indistinguishable phenotype when grown in pots without salt stress (**Figure 5B**). As per literature, this appears to be the highest level of salt tolerance for tobacco, the plant with broader leaves and with more exposed surface area for transpiration.

### Drought Tolerance and Chlorophyll Contents in Transplastomic Plants

**FIGURE 6:**
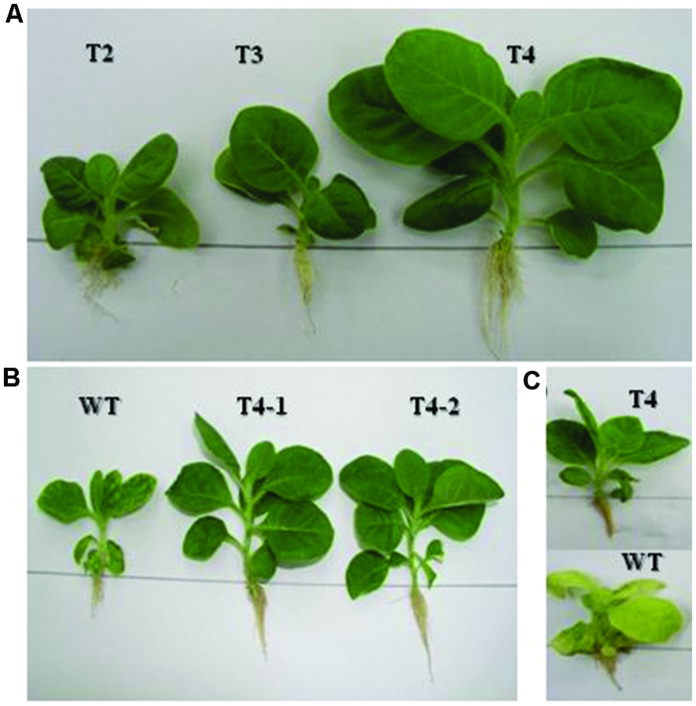
**Drought tolerance assays are performed on transplastomic plants in liquid MS medium that carries increasing levels (1–6%) of PEG. (A)** Shows growth of primary plants clone at 5% PEG in the medium. Whereas **(B)** shows growth of representative plants from T4 clone at 5% PEG in the medium. In **(C)** representative transgenic and WT plants are growing at 6% PEG with distinguished phenotypes.

The homoplasmic transgenic plants exhibiting high enzyme activity and salt tolerance were tested for drought tolerance. Seeds from transgenic and WT plants were first germinated on solidified MS medium because seeds cannot be germinated in liquid medium. Then, 7 day-old seedlings were transferred to MS liquid medium containing increasing levels of polyethylene glycol, ranging from 1 to 6% in Magenta boxes, carrying plastic bridges to hold seedlings under *in vitro* conditions. As shown in **Figure 6A**, chloroplast transgenic seedlings grew variably in 5% PEG containing liquid MS medium in Magenta Boxes. Variable growth of plants was due to the genetically inconsistent (heteroplasmic) nature of plants. The inconsistent growth of heteroplasmic plants compared with uniform growth of homoplasmic plants in 5% PEG containing liquid MS medium clearly show that the increasing concentrations of the ARDH are advantageous to normal growth of transgenic plants under stress (**Figure 6B**). Transgenic T4 plant stayed green and developed as a normal plant in MS liquid medium that was supplemented with 6% PEG whereas control plant exhibited loss of chlorophyll and growth retardation, ultimately died (**Figure 6C**). Loss of chlorophyll in WT plant indicates breakdown of chloroplast thylakoid membranes due to osmotic stress caused by PEG. Whereas presence of chlorophyll in transgenic chloroplasts indicates the integrity of thylakoid membranes, even in the presence of very high concentration (6%) of PEG (**Figure 6**), clearly demonstrating the benefit of the expressed gene in transgenic chloroplasts. It was impossible to measure and compare the chlorophyll contents of transgenics with WT plants under stress conditions because WT plants bleached on PEG containing medium. The WT and transgenic plants were phenotypically indistinguishable in pots when grown without stress (**Figure 5B**).

## Discussion

Osmoprotectant concentrations to the levels of 200 mM or more are osmotically significant; subsequently have essential role in water uptake under stress by maintaining cell turgor as well as the driving gradient (Rhodes and Samaras, 1994). Amongst osmoprotectants reported in the literature, glycine betaine, mannitol, and proline occur commonly in plants, whereas trehalose occurs rarely in plants but arabitol is found only in bacteria and yeast. Enzymes responsible for most of these and some other compounds, used to engineer plants for stress tolerance, have been studied under laboratory conditions. Under stress conditions, glycine betaine protects the sub-cellular structures, transcriptional, and translational machineries and also intervenes in the refolding of enzymes as a molecular chaperone (Wani et al., 2013). In our studies we have expressed an NAD-dependent D-*ArDH* gene from *Candida albicans* in chloroplasts to develop tolerance in tobacco to environmental stresses, for example salinity and drought. As *ArDH* reduced NAD in the presence of arabitol and chloroplasts remained green due to the accumulation of chlorophyll under stress hence, it is likely that salinity and drought tolerance in transgenic plants is due to the developed arabitol. Further, green color and normal growth of plants under stress conditions clearly demonstrate the customary functioning of transgenic plant chloroplasts. Hence, this is the first report to express *ArDH* gene in plant chloroplasts that reduces D-ribulose into D-arabitol. An *E. coli* gene, atlD, that converts non-plant-metabolizable arabitol into metabolizable xylulose in the medium, was expressed in rice in order to develop a positive selection system (Lafayette et al., 2005) since rice cannot metabolize D-arabitol (Stein et al., 1997). In another report, betaine aldehyde dehydrogenase gene was expressed to accumulate betaine in carrot plastids where improvement in water stress tolerance has been claimed (Kumar et al., 2004) since betaine occur commonly in plants.

**FIGURE 7:**
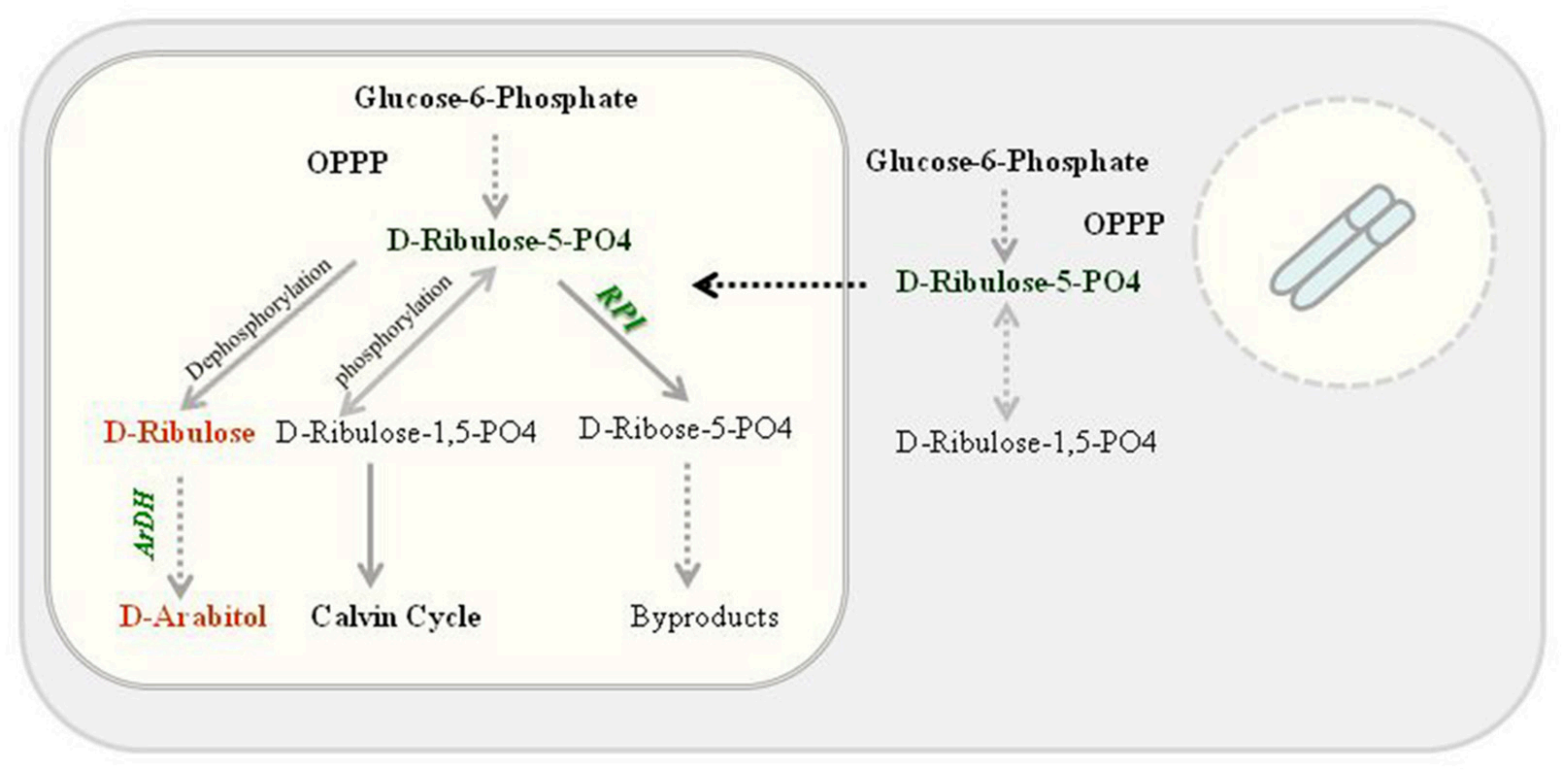
**Proposed D-arabitol pathway in tobacco chloroplasts.** ARDH reduces D-ribulose to D-arabitol where D-ribulose is derived by dephosphorylating D-ribulose-5-PO4 in the oxidized pentose pathway. Nevertheless, phosphorylation of D-ribulose-5-PO4 results in D-ribulose-1,5-bisPO4 that enters into Calvin cycle.

High ARDH enzyme activity was observed in fully expanded leaves of potted plants (T4 plants) compared to primary transgenic plants when exposed to continuous light as *psbA* 5′ UTR is light regulated. Transgenically it was confirmed that accumulation of transprotein is in response to light and is controlled via *cis*-acting regulatory elements in the untranslated region of the psbA mRNA because reduction in accumulation was observed when these plants were transferred to dark but no reduction in mRNA levels was observed (Staub and Maliga, 1993). Transprotein accumulation was recorded as high as 135- to 200-fold (Staub and Maliga, 1994) in response to light, however, deletion of sequences from 5′ UTR were resulted in fourfold decrease in translational efficiency (Eibl et al., 1999) but no change in translational efficiency was observed after exchanging the *psbA* 3′ UTR (Staub and Maliga, 1994; Eibl et al., 1999), only mRNA levels were decreased, confirming that the *psbA* 5′ UTR is light responsive (Eibl et al., 1999). We expressed *ArDH* gene under *psbA* promoter along with 5′ UTR and an enhanced enzyme activity was observed. Though substrate specificity is unknown yet in plants but based on yeast, it is likely that ARDH reduces D-ribulose to D-arabitol where D-ribulose is derived by dephosphorylating D-ribulose-5-PO4 in the oxidized pentose pathway in plant chloroplasts. But phosphorylation of D-ribulose-5-PO4 results in D-ribulose-1,5-bisPO4 that enters into Calvin cycle (**Figure 7**).

When transplastomic plants were observed tolerant to very high levels of NaCl (300–350 mM) and were green in color without any pleiotropic effects, then 7 day-old transplastomic and non-transformed control seedlings were grown in PEG-containing liquid MS medium. The transplastomic seedlings grew normal whereas control seedlings exhibited loss of chlorophyll and growth retardation. Loss of chlorophyll in WT plants indicates breakdown of chloroplast thylakoid membranes due to osmotic stress induced by PEG. But the presence of chlorophyll in transgenic chloroplasts indicates the integrity of thylakoid membranes, even in the presence of high concentrations of PEG, clearly demonstrating the advantage of expressed gene in transgenic chloroplasts.

Accumulation of arabitol in chloroplasts is not measured this, then, is one aspect that calls for further investigation. Yet, it is believed that accumulation in smaller quantities is adequate to protect chloroplasts from salt and drought stresses and larger quantities are advantageous for commercial applications.

## Materials and Methods

### Plant Material and Growth Conditions

*Nicotiana tabacum*, var. Petit Havana, was grown aseptically on 0.7% phyta-agar-solidified MS salts, pH 5.8, containing 3% sucrose at 25°C ± 02 under 100°μmole photons m^-2^ s^-1^ (16 h light, 8 h dark). Fully expanded leaves of 4–6 week-old plants were used for chloroplast transformation using microprojectile bombardments.

### Development of Chloroplast Transformation Vector

To develop transgenic chloroplasts, transformation vector was developed as described (Khan et al., 2007). The flanking sequences were amplified using primers 5′-GATATCAAAACCCGTCCTCAGTTCGGATTGC-3′ and 5′-GATATCCACGAGTTGGAGATAAGCGGA-3′). The underlined sequences are the created *Eco*RV sites, which were used to restrict the cloned PCR product from the TA cloning vector (MBI Fermentas, Italy) and to ligate it into the pBluescript II (MBI Fermentas, Italy) plasmid, opened with PvuII restriction enzyme. An adopter carrying unique restriction sites was ligated into the insertion site (PvuII) for subsequent cloning of selection and expression cassettes. The *psbA* promoter and 5′UTR were amplified using primers 5′AAGCTTACTAGCATATCGAAATTCT-3′ and 5′GAATTCCATATGAAAATCTTGGTT-3′ and the PCR-amplified fragment was cloned into TA cloning vector (MBI Fermentas, Italy). The *psbA* 3′UTR was amplified using primers, 5′-TCTAGAATCTAGATCGTGC-3′ and 5′-GAGCTCGGTGACCCTTGTATG-3′ and cloned downstream of the promoter and 5′UTR of *psbA*. The *ArDH* gene was amplified from *Candida albicans* and cloned into the *psbA* cassette. The expression cassette carrying *ArDH* gene was cloned downstream to *aadA* gene that is tethered with 16SrRNA operon promoter to generate the final transformation vector, pMSK83. Hence, both genes were expressed under separate promoters, independently but sharing the terminating sequence, 3′UTR of *psbA*.

### Chloroplast Transformation and Selection of Transgenics

The transformation vector pMSK83 was quoted onto the surface of 0.6 μ gold particles to transform tobacco chloroplast using optimized protocols (Svab and Maliga, 1993; Khan and Maliga, 1999; Daniell et al., 2002; Bock and Khan, 2004). Fully expanded tobacco leaves from 4 to 6 week-old plants were used and the transformation was carried out using a PDS 1000 helium gun (BIO-RAD). Bombarded leaves were chopped into small pieces of 3 mm × 3 mm size after 48 h of bombardments, which were placed on spectinomycin (500 mg/l)-containing RMOP medium (Svab and Maliga, 1993; Khan and Maliga, 1999). Spectinomycin resistant shoots were recovered from bleached leaf pieces on RMOP medium (Khan and Maliga, 1999) and rooted on solidified MS medium (Murashige and Skoog, 1962).

### Genomic Analysis of Transgenic Plants to Determine the Homoplasmy

Total cellular DNA from WT as well as transgenic plants was isolated using the hexadecyltrimethyl ammonium bromide (CTAB) DNA extraction method (Rogers and Bendich, 1985) with modifications and was used as template in PCR reactions. The integration of transgenes carrying selection and expression cassettes into the plastome was confirmed using *aadA*- and flanking sequences-specific primers and probes, as described in results section. The homoplasmy for transgenes integration was confirmed by Southern blotting using flanking sequences as a probe.

### Enzyme Assays

Arabitol dehydrogenase enzyme assay was carried out as described by Wong et al. (1993) with modifications where standard curve was developed by using different concentrations of BSA and the standard factor was calculated as 0.01 μg/mL/min. Crude extracts were prepared from plants that were grown under *in vitro* conditions at 25°C ± 02 and 100 μmole photons m^-2^ s^-1^ (16 h light, 8 h dark) as well as from plants that were kept under continuous light for 7 days. The crude extracts were prepared by homogenizing 0.25 g tissues from fourth fully expanded leaf (from top to bottom) on ice (at ~04°C) in 500 μl homogenization buffer. The buffer contains 35 mM HEPES (*N*-2-hydroxyethylpiperazine- *N*9-2-ethanesulfonic acid; pH 8.5), 0.2 mM NAD or NADP, 0.2 mM phenazine methosulfate, 0.4 mM MTT and 12.5 mM D-arabitol. Spectrophotometer was used to measure the reduction of 3-(4,5-dimethylthiazol-2-yl)-2,5-diphenyl-tetrazolium bromide (MTT) at wavelength (*A*578). The enzyme activity was calculated in μmoles/ml/min (The amount of the enzyme necessary to convert 1 μmole of substrate per min at 25°C) as described by Wong et al. (1993, 1995).

### Salinity and Drought Tolerance Assays

Nodal segments and seeds from transgenic and WT plants were subjected to various levels of NaCl (Sigma) and polyethylene glycol (MW, 8000, Sigma). Plants developed from nodal segments were variable in size hence seeds from transgenic and WT plants were tested on solidified MS medium supplemented with increasing intensities (100–600 mM) of NaCl. Seedlings were raised from seeds of both transgenic and non-transgenic WT plants to grow in liquid MS medium in magenta boxes under standard light–dark conditions. The MS medium was supplemented with different concentrations (1–6%) of polyethylene glycol. The seedlings subjected to shock episodes were analyzed for stress tolerance.

## Conflict of Interest Statement

The authors declare that the research was conducted in the absence of any commercial or financial relationships that could be construed as a potential conflict of interest.

## Abbreviations

*ArDH*: arabitol dehydrogenase
NaCl: sodium chloride
PEG: poly ethylene glycol.
